# Type VI secretion drives reciprocal growth inhibition among symbiotic Vibrio fischeri strains

**DOI:** 10.1099/mic.0.001781

**Published:** 2026-10-23

**Authors:** Anjali E. McNeil, Landon B. Teeter, Jaedon I. Burkhart, Nathaniel J. Flores, Yusuf A. Shabana, Elizabeth J. Giacobe, Peyton J. Moore, Justice P. Jensen, Aidan R. Donnelly, Andrew N. Murtha, Harun Aslan, Andrew G. Cecere, Kirsten R. Guckes, Tim I. Miyashiro

**Affiliations:** 1 Department of Biochemistry and Molecular Biology, Pennsylvania State University, University Park, PA, USA; 2 The One Health Microbiome Center, Huck Institutes of the Life Sciences, Pennsylvania State University, University Park, PA, USA; 3 Department of Biological Sciences, Salisbury University, Salisbury, MD, USA

**Keywords:** genetics, symbiosis, toxin, T6SS

## Abstract

Bacterial cells are often in direct contact with one another and compete for nutrients and space. The type VI secretion system (T6SS) is a contact-dependent mechanism that enables bacteria to deliver toxic effectors into neighbouring cells. *Vibrio fischeri* is a bacterial symbiont of the Hawaiian bobtail squid *Euprymna scolopes* and encodes a T6SS on its second chromosome (T6SS2) that promotes interference competition among strains within the squid light organ. Effector diversity among T6SS2^+^ strains is poorly understood, which impedes predicting compatibility among strains within the light organ. Here, we report our investigation of strain ANM004 and how T6SS2 facilitates its interactions with other strains including the model T6SS2^+^ strain FQ-A001. We show that both strains exhibit reciprocal T6SS2-dependent antagonism. Comparative genomics reveals each strain encodes unique effectors within T6SS2 gene loci. Heterologous expression of an ANM004-encoded AHH nuclease effector in FQ-A001 inhibits kin growth in a T6SS2-dependent manner. This effect on FQ-A001 kin is abrogated by expression of the cognate immunity factor encoded downstream of the effector gene. Together, these results establish an experimental framework for investigating intercellular interactions between two T6SS2^+^ symbiotic strains.

## Data Summary

The following sequence data are deposited in GenBank under BioProject PRJNA1453452 with BioSample accessions: Strain ANM004 genome: SAMN57263641; Oxford Nanopore sequence data of ANM004 genome: SRR38098049; Illumina paired-end sequence data of ANM004 genome: SRR38098050.

## Introduction

Bacteria commonly inhabit densely populated environments among other cells, creating intense competition for limited resources and space. Consequently, many species have evolved mechanisms of interference competition [1, 2], i.e. the antagonistic strategies that inhibit or eliminate neighbouring competitors. Elucidating the molecular basis of these interactions is important for understanding how bacteria engage with one another within complex communities and, ultimately, for identifying strategies to manipulate the structure and function of microbial ecosystems.

One mechanism that promotes interference competition is the type VI secretion system (T6SS), which is a cytoplasmic, contractile nanomachine anchored to the cell envelope and oriented outward [2]. It functions as a molecular lancet, propelling a needle-like structure to deliver toxic effector proteins directly into neighbouring cells. Target cells that are susceptible to these effectors may undergo lysis or experience growth inhibition. An emerging platform to study the ecological impact of T6SS involves the Gram-negative, bioluminescent bacterium *Vibrio fischeri* (also known as *Aliivibrio fischeri*) [3]. *V. fischeri* forms dense, light-emitting populations within the light organ of the Hawaiian bobtail squid (*Euprymna scolopes*), which uses this bioluminescence for counterillumination [4]. All *V. fischeri* strains encode components of a T6SS on their first chromosome, referred to as T6SS1, but the biological significance of this system has yet to be realized. A subset of strains, including FQ-A001, additionally encode a second T6SS on their second chromosome, referred to as T6SS2 [3].

Over the past decade, studies of FQ-A001 have implicated T6SS2 in mediating interference competition among strains co-colonizing the same site within the squid light organ [5–7]. When an FQ-A001 cell enters a colonization site, it will exhibit antagonism towards other cells, often eliminating those susceptible to T6SS2-based attacks [8]. The bacterial enhancer binding protein VasH promotes σ^54^-dependent expression of T6SS2 structural factors such as the needle subunit Hcp, which is encoded by *hcp* and *hcp1* genes [9, 10]. As the population grows, cells engage in quorum sensing, i.e. the intercellular signalling mechanism based on detection of self-produced small diffusible signalling molecules. At high cell density (HCD), quorum sensing triggers bioluminescence production [4] and transcriptionally shuts down T6SS2 as indicated by lower transcriptional expression of *hcp* genes [7]. Lower *hcp* expression is mediated by transcriptional repression of *vasH* by the quorum-sensing regulator LitR [7], which is stabilized at HCD [11].

Previous work has suggested that more than half of symbiotic *V. fischeri* strains encode a T6SS2 [8]. However, most studies investigating the molecular basis of T6SS2 activity have focused on their interactions with T6SS2^-^ strains. As a result, understanding of effector diversity in *V. fischeri* remains limited, due in part to a reliance on preliminary bioinformatic analyses [6] that have constrained deeper functional and ecological characterization of T6SS2. Here, we investigate ANM004, a T6SS2^+^ strain predicted to encode effectors distinct from those of the canonical T6SS2^+^ strain FQ-A001. By establishing a system involving two T6SS2^+^ strains, we provide new insight into how interference competition shapes the ecology of this bacterial symbiont.

## Methods

### Media and growth conditions

*V. fischeri* strains (Table 1) were grown aerobically in Luria-Bertani salt (LBS) medium [1% (wt/v) tryptone, 0.5% (wt/v) yeast extract, 2% (wt/v) NaCl, 50 mM Tris/HCl (pH 7.5)] or seawater-tryptone with osmolarity (SWTO) medium [0.5% (wt/v) tryptone, 0.3% (wt/v) yeast extract, 0.3% of glycerol in artificial seawater [12]]. Solid media was supplemented with 1.5% wt/v agar. Plasmids were maintained using a concentration of 2.5 µg ml^−1^ chloramphenicol or 100 µg ml^−1^ kanamycin.

**Table 1.** Strains and plasmids used in this study

Strain	Genotype	Reference
ES114	Wild-type (WT) symbiotic *V. fischeri* strain	[27]
FQ-A001	WT symbiotic *V. fischeri* strain	[5]
ANM004	WT symbiotic *V. fischeri* strain	[6]
TIM313	ES114 Tn*7*::*erm*	[11]
AGC020	FQ-A001 Tn*7*::*erm*	[28]
TIM426	ANM004 Tn*7*::*erm*	This study
KRG001	FQ-A001 Δ*litR*	[7]
KRG014	FQ-A001 Δ*hcp* Δ*hcp1* Δ*litR*	This study
KRG017	ANM004 Δ*tssF*	This study
NPW58	FQ-A001 Δ*hcp* Δ*hcp1*	(Guckes 2019)
**Plasmid**	**Relevant genotype**	**Reference**
pSNS119	P*_lac_*::*tssB*::*gfp*	[6]
pEVS79	Shuttle vector	[29]
pKRG037	pEVS79 Δ*tssF*	This study
pTM214	P*_trc_*::*mCherry*	[30]
pAEM002	P*_trc_*:: *AC1TBE_15825-30*	This study
pKRG055	P*_trc_*:: *AC1TBE_15825*(H38A)*-30*	This study
pSCV51	P*_D_*::*gfp*	[31]
pTM447	P*_D_*:: *AC1TBE_15830*	This study
pTM464	P_*D*_:: *AC1TBE_15875*	This study

*Squid colonization assays:* Hatchling *E. scolopes* were generated using the mariculture facility as previously described [13]. To initiate a squid colonization assay, starter cultures of *V. fischeri* strains grown in LBS were diluted 1 : 100 into fresh LBS medium and grown to OD_600_=1.0. Cultures were diluted into filter-sterilized seawater (FSSW) and combined with hatchlings so that the inoculum was ~5,000 c.f.u. ml^−1^. After 3.5 h, animals were washed and transferred to vials containing 4 ml FSSW. At 24 h post-inoculation, bioluminescence levels were measured using a GloMax 20/20 and animals were frozen at −80 °C. Bacterial abundance was determined by plating out animal homogenate onto LBS and enumerating c.f.u.

*Molecular biology:* Plasmid pAEM002 expresses *AC1TBE_15825-30* from the IPTG-inducible *trc* promoter (P*_trc_*). Genes *AC1TBE_15825-30* were amplified from genomic DNA of ANM004 using primers pTrc-AHH-IF-*Kpn*I-u1 and -*Sal*I-l1 (Table 2) and PFU Ultra Polymerase according to the manufacturer’s instructions. The amplicon was cloned into pCR-Blunt. A 1.4 kb fragment containing *AC1TBE_15825-30* was excised from the pCR-Blunt clone using *Kpn*I/*Sal*I and cloned into pTM214 using the restriction enzymes *Kpn*I/*Sal*I, which excises *mCherry*.

**Table 2.** Primers used in this study

Primer name	Primer sequence (5′→3′)
pAEM002 construction	
pTrc-AHH-IF-*Kpn*I-u1	ATCGCAGGTACCATGCCAATTAGAGAAAATGAAGGT
pTrc-AHH-IF-*Sal*I-l1	TAGGCTGTCGACTTAGAATTTAGGAGCTCGAACACT
pKRG055 construction	
ANM_AHH_H38AF	AGTTGCATCACACGCAATAATTTCATGTGA
ANM_AHH_H38AR	TCACATGAAATTATTGCGTGTGATGCAACT
pTM447 construction	
ANM004_IF-proD-*Xba*I-u1	GGTCTAGAGATTATTGAATATTCGAGAACAAA
ANM004_IF-proD-*Xho*I-l1	GGCTCGAGAGAGAATATGGTATTCATTTTTCA
pTM464 construction	
ANM004_tssF-proD-*Spe*I-u1	GGACTAGTGGTCAATTTAATGTGTAGTAGGAG
ANM004_tssF-proD-*Xho*I-l1	GGCTCGAGGAATCATAATATAGTTTGCTGCCC
pKRG037 construction	
ANM004-tssF-del-5-*Kpn*I-u2	GGGGTACCGGTCCACAAAGTGGTGGTACAGTA
ANM004-tssF-del-5-*Xba*I-l2	GGTCTAGAGCTCACTATTAACTCCTACTACACA
ANM004-tssF-del-3-*Xba*I-u2	GGTCTAGAGAAGTGATAGATGAACATACTGG
ANM004-tssF-del-3-*Sac*I-l2	GGGAGCTCATTTTCGTCTCCTCCCCTTTTG

Plasmid pKRG055 is similar to pAEM002 but expresses *AC1TBE_15825* H38*A-30*. To generate the H38A allele of *AC1TBE_15825*, site-directed mutagenesis was conducted on the pCR-Blunt clone described above using primers ANM_AHH_H38AF and R (Table 2) and Phusion polymerase. The reaction product was subsequently subjected to DpnI treatment and transformed into TOP10 *Escherichia coli* cells. Mutations were validated by sequencing, and the strategy described above was used to subclone the insert into pTM214.

Plasmid pTM447, which contains *AC1TBE_15830* constitutively expressed from a synthetic promoter (P*_D_*), was constructed by amplifying *AC1TBE_15830* from genomic DNA of ANM004 using primers ANM004_IF-proD-*Xba*I-u1 and -*Xho*I-l1 (Table 2) and PFU Ultra Polymerase according to the manufacturer’s instructions. The amplicon was cloned into pSCV51 using restriction enzymes *Xba*I/*Xho*I, which excises a 5′ fragment of *gfp*. Plasmid pTM464, which contains *AC1TBE_15875* constitutively expressed from P*_D_*, was constructed in a similar fashion but using primers ANM004_tssF-proD-*Spe*I-u1 and -*Xho*I-l1 (Table 2) .

The deletion allele Δ*tssF*, which replaces codons 3–559 of *tssF* (*AC1TBE_15875*) with Ser-Arg codons encoded by an *Xba*I site, was generated using ANM004 genomic DNA by PCR amplification of 1 kb regions flanking 5′ and 3′ ends of *AC1TBE_15875* with primer sets ANM004-tssF-del-5-*Kpn*I-u2 and -*Xba*I-l2 and ANM004-tssF-del-3-*Xba*I-u2 and *Sac*I-l2 (Table 2) . The fragments were cloned into pEVS79 shuttle vector using *Kpn*I/*Sac*I, resulting in pKRG037. To generate the knockout mutant, pKRG037 was introduced into ANM004 by conjugation and screening by PCR for the double-crossover event, according to a previously established protocol [11].

*TssB-GFP localization assay*: Starter cultures of strains harbouring pSNS119, encoding a *tssB::gfp* translational fusion under control of an IPTG-inducible *lac* promoter, were grown overnight at 28 °C in LBS medium supplemented with kanamycin. Starter cultures were diluted 1 : 100 into fresh LBS medium containing kanamycin ±0.5 mM IPTG. After ~4 h, 3 µl of induced culture was spotted onto a clean glass coverslip. Cells were immobilized by gently overlaying the suspension with a 1.5% (wt/v) agar plug prepared in LBS medium containing kanamycin ±IPTG. The agar plug was flattened and secured by placing a microscope slide on top. GFP fluorescence and differential interference contrast images were acquired using a Zeiss LSM 780 NLO confocal microscope equipped with a 63× water objective. GFP was excited at 488 nm. The pinhole was set to fully open. Imaging parameters (laser power, gain, exposure) were kept constant across samples. Images were exported as eight-bit TIFF files and cropped using ImageJ.

*Comparative genomics:* To sequence the genome of ANM004, the genomic DNA was isolated from 0.5 ml of a LBS starter culture of ANM004 using the MasterPure Complete DNA and RNA Purification Kit (EpiCentre). The genomic DNA was sequenced using a hybrid approach combining long-read sequencing from Nanopore (ONT) and short-read sequencing from Illumina (SeqCenter, LLC). Illumina sequencing libraries were prepared using the tagmentation-based and PCR-based Illumina DNA Prep Kit and custom IDT 10 bp unique dual indices with a target insert size of 320 bp. No additional DNA fragmentation or size-selection steps were performed. Sequencing files are deposited in NCBI Sequence Read Archive under BioProject PRJNA1453452.

To identify the locations of T6SS2 and auxiliary gene clusters in ANM004, the blastx-2sequences option in NCBI was used with the ANM004 genome query and Hcp and VgrG homologues in FQ-A001 as subject. To identify homologues of predicted proteins of ANM004 in FQ-A001, blastp-2sequences option in NCBI was used with putative T6SS2 protein sequences of ANM004 as query and the translated cds file associated with FQ-A001 (NCBI RefSeq assembly GCF_004359415.1) as subject. Conserved domains were identified using the Batch CD-Search Tool in NCBI with Concise Data mode and Include domain defline options selected.

*Co-incubation assays:* Co-incubation assays were performed using a protocol similar to that previously described [9]. Starter cultures of the indicated strains grown in LBS medium were normalized to OD_600_ of 1.0 in a final volume of 1 ml using LBS as the diluent. For each strain combination, 50 µl volumes of the normalized cell suspensions were combined in a microcentrifuge tube and briefly vortexed. To initiate the assay, a 10 µl volume of the resulting cell mixture was spotted onto the surface of LBS agar, with no more than six distinct samples per Petri dish, and incubated at 24 °C. When appropriate, plates also contained 1 mM IPTG for induction of the P*_trc_* promoter. Biological replicates were staggered by 30 min and performed on separate Petri dishes.

For experiments involving different cell densities, normalized starter cultures were used to inoculate fresh SWTO medium at 1 : 1,000 or 1 : 10,000. Cultures grew at 28 °C with shaking until cultures reached an OD_600_ of ~2.0 and 0.20, respectively. Cultures were normalized to an effective OD_600_=1.0 by centrifugation and combined with the starter culture of the erm^R^ strain as described above.

To quantify total and erythromycin-resistant (erm^R^) populations within each spot, an agar plug encompassing the entire spotted area was excised using flame-sterilized forceps and suspended in 1 ml LBS medium. The suspensions were serially diluted, and 0.1 ml volumes were plated onto LBS agar ±erm. Plates were incubated at 24 °C and c.f.u. were enumerated. Relative abundance was calculated as the ratio of erm^R^ c.f.u. to total c.f.u.

*Growth assays:* Starter cultures were normalized to OD_600_=1.0 and then diluted 1/100 into 0.1 ml of LBS or SWTO in a 96-well plate. Plates were incubated at 28 °C with shaking (240 rpm) in a Spark Cyto plate reader (Tecan). Absorbance at 600 nm was measured every 30 min.

*Structural predictions:* All structural predictions were generated using the AlphaFold 3.0 Server with default parameters [14]. Two Zn^2+^ ions were included with queries involving AC1TBE_15825. Models were further formatted and visualized for figures using ChimeraX v.1.8 [15].

## Results

### ANM004 is a T6SS2+ symbiotic strain of *V. fischeri*

FQ-A001 is a model strain for elucidating the molecular mechanisms underlying the T6SS2 of *V. fischeri* [3]. To begin investigating intercellular interactions between T6SS2^+^ *V. fischeri* strains, we first identified another T6SS2^+^ symbiotic strain to pair with FQ-A001. Strain ANM004 was originally isolated from an adult male squid (mantle length: 2.7 cm) collected from Maunalua Bay, HI in May 2015. Exposure of hatchling squid to seawater containing ANM004 results in animals that produce bioluminescence and harbour c.f.u. at levels higher than the type strain ES114 (Fig. 1), indicating that ANM004 is a bioluminescent strain of *V. fischeri* that can establish symbiosis with *E. scolopes*.

**Fig. 1.**
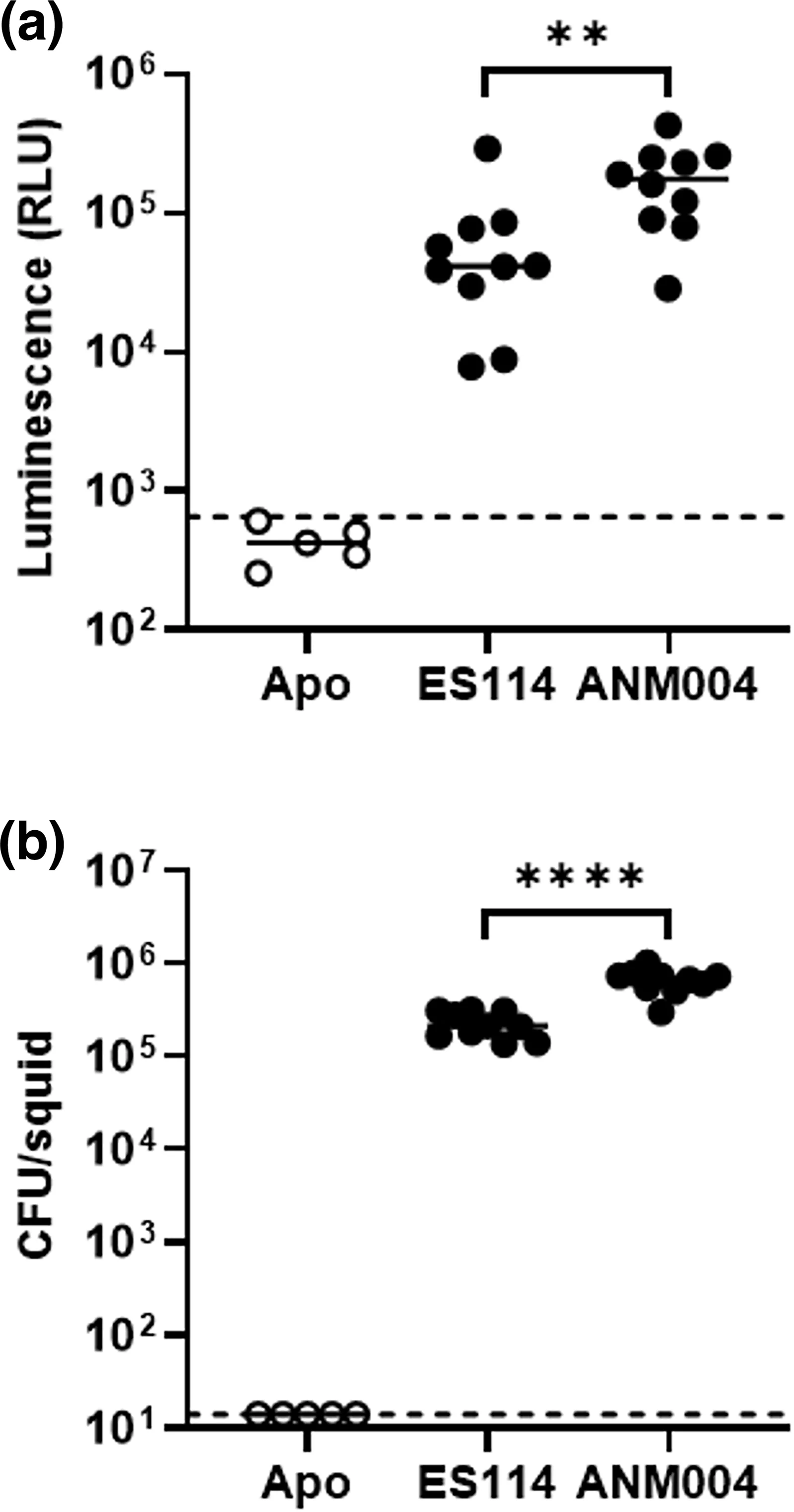
ANM004 is a symbiotic strain of *V. fischeri.* (**a)** Bioluminescence measurements of hatchlings (*N*=10) 24 h after a 3.5 h exposure to seawater containing no bacteria (Apo) or inoculated with strains ES114 or ANM004. The dotted line indicates the 95th percentile of bioluminescence in the Apo group and serves as the threshold for scoring animals as bioluminescent. Closed (open) symbols indicate (non-)bioluminescent animals. *P*-value was determined using an unpaired *t*-test with Welch’s correction on log-transformed data (**: *P*=0.0073). (**b)** Abundance of *V. fischeri* in the animals shown in panel a, measured 24 h post-inoculation. The dotted line indicates the limit of detection (14 c.f.u.). Closed (open) symbols indicate (not) colonized. *P*-value was determined using an unpaired *t*-test with Welch’s correction (****: *P*<0.0001).

Having demonstrated that ANM004 can establish symbiosis, we next used *in vitro* assays to examine traits associated with T6SS2. One of our previous studies reported that ANM004 contains a genetic marker consistent with the *T6SS2* gene locus and can inhibit the growth of the T6SS2^-^ strain ES114 [6]. To determine whether T6SS2 promotes aggression in ANM004 towards ES114, we first generated a *tssF* (*AC1TBE_15875*) mutant, which cannot express an essential component of the T6SS2 baseplate and grows similarly to WT (Fig. S1, available in the online Supplementary Material). When harbouring a construct that expresses sheath protein TssB fused to GFP (TssB::GFP), the *tssF* mutant fails to form the subcellular sheath structures visualized as GFP puncta observed in wild-type (WT) cells (Fig. 2a), confirming the critical role TssF plays in T6SS2 assembly. To test whether T6SS2 impacts ANM004 antagonism towards ES114, we then performed a co-incubation assay in which ES114 was marked with erythromycin resistance (erm^R^) to track its abundance. Such co-incubation assays reveal whether the presence of another strain impacts the normal growth of the marked strain, providing insight into interstrain interactions. The abundance of erm^R^ ES114 decreased 10-fold during co-incubation with ANM004 (Fig. 2b), whereas with the *tssF* mutant it grew normally by increasing 7.6-fold, suggesting ANM004 aggression towards ES114 depends on its T6SS2. Expression of *tssF* from a plasmid in the Δ*tssF* mutant restored its ability to inhibit erm^R^ ES114 (Fig. 2b), demonstrating genetic complementation. Taken together, these results establish ANM004 as a T6SS2^+^ symbiotic strain of *V. fischeri*.

**Fig. 2.**
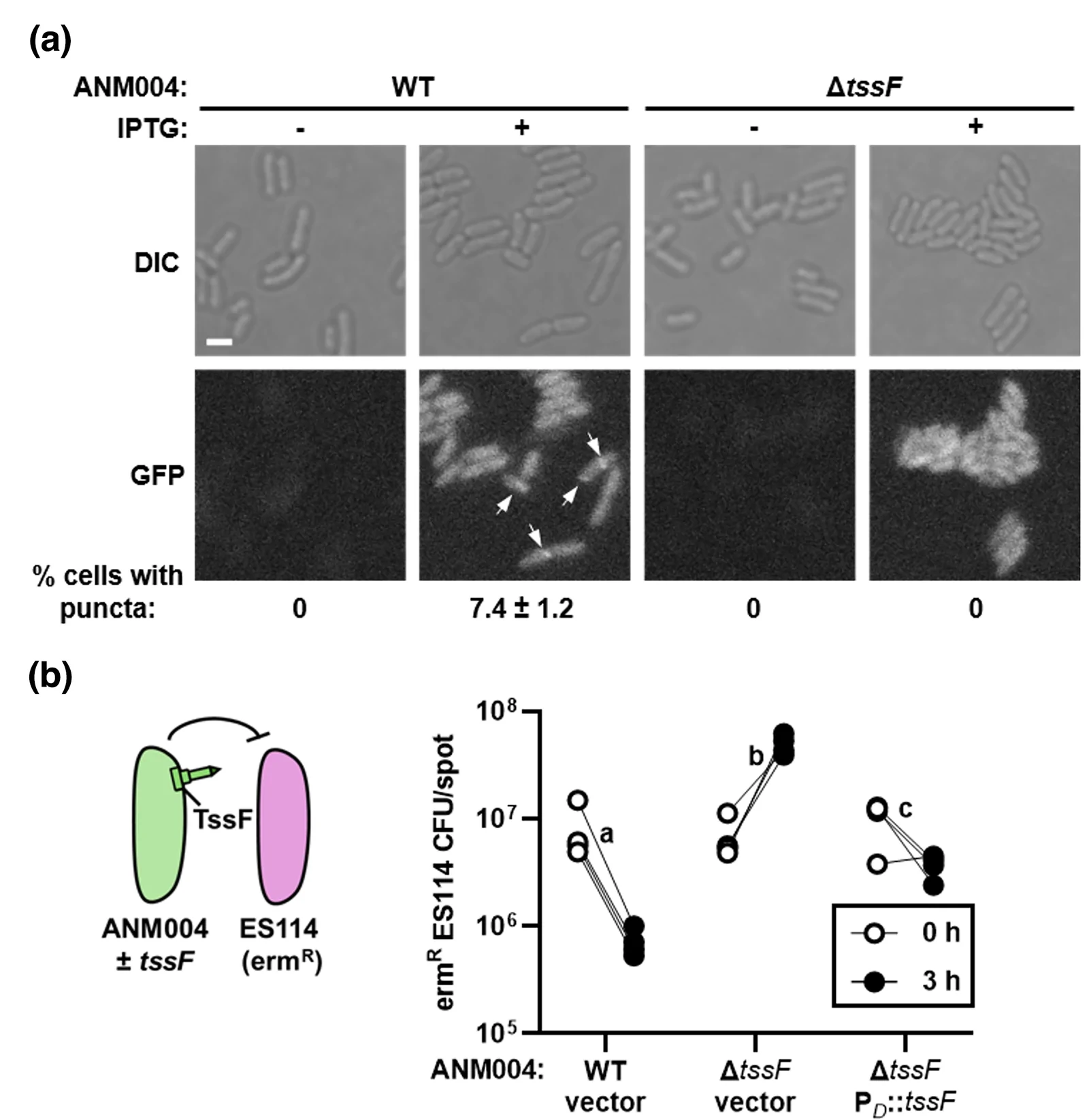
ANM004 exhibits T6SS2-dependent aggression towards ES114. (**a)** TssB::GFP localization pattern in ANM004 (WT) and KRG017 (Δ*tssF*) harbouring pSNS119 plasmid and grown in LBS±0.5 mM IPTG. Arrows point to examples of subcellular GFP puncta. The percentage of cells with puncta (avg±SD) was determined by counting at least 200 cells associated with independent cultures (*N*=3). Scale bar=2 µm. (**b)** *Left*, cartoon of model of T6SS-dependent interactions between ANM004 and ES114. *Right*, abundance of ES114-derived erm^R^ strain TIM313 (erm^R^) during co-incubation with ANM004 (WT) or KRG017 (Δ*tssF*) harbouring pSCV51 (vector) or pTM464 (P_D_::*tssF*). *N*=4. Different letters indicate groups with statistically different log-transformed fold-changes in abundance over time (*P*<0.05, adjusted for multiple comparisons), as determined by one-way ANOVA (F_2,9_=103.2, *P*<0.0001).

### Interference competition between ANM004 and FQ-A001

Previous studies of T6SS2 activity in FQ-A001 involved ES114-derived strains [3], so how it interacts with different T6SS2^+^ strains is poorly understood. To investigate interactions between ANM004 and FQ-A001, we performed co-incubation assays in which one strain was marked erm^R^ to track its abundance. After 3 h of co-incubation with ANM004, the abundance of erm^R^ FQ-A001 decreased 6.0-fold (Fig. 3a), suggesting that ANM004 exhibits antagonism towards FQ-A001 that results in their death. Furthermore, erm^R^ FQ-A001 grew 3.1-fold when co-incubated with the *tssF* mutant (Fig. 3a), indicating that this antagonism depends on the T6SS2 of ANM004.

**Fig. 3.**
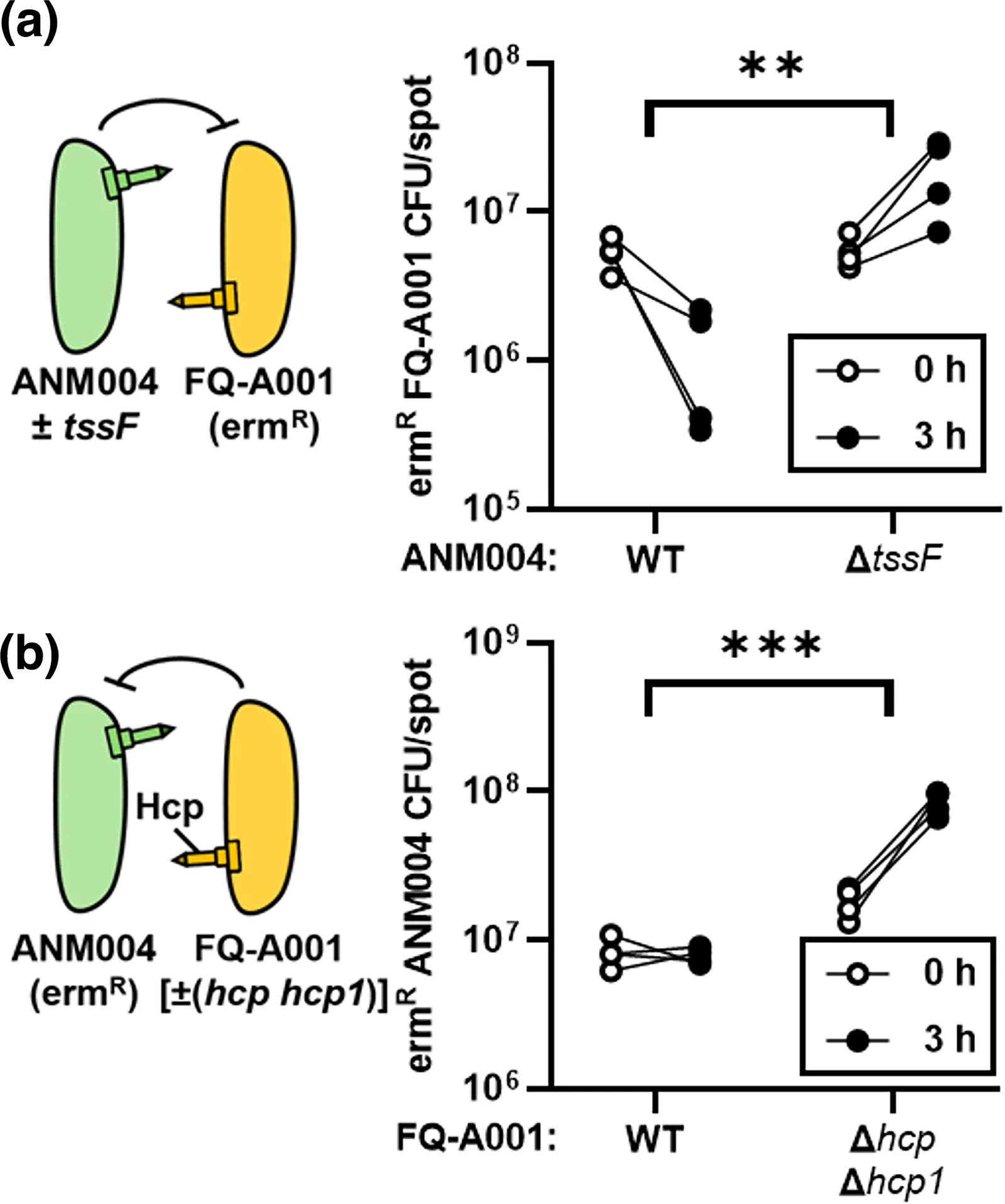
Reciprocal T6SS2-dependent aggression between ANM004 and FQ-A001. (**a)** Abundance of erm^R^ FQ-A001-derived strain AGC020 (FQ-A001 Tn*7::erm*) co-incubated with ANM004 (WT) or KRG017 (Δ*tssF*) at indicated time points. *N*=4. **: *P*=0.0055. (**b)** Abundance of erm^R^ ANM004-derived strain TIM426 (ANM004 Tn*7::erm*) co-incubated with FQ-A001 (WT) or NPW58 (Δ*hcp* Δ*hcp1*) at indicated time points. *N*=4. ***: *P*=0.0004. *P*-values were calculated by Welch’s two-tailed *t* tests on log-transformed fold changes in abundance over time between groups.

To determine how FQ-A001 affects the abundance of ANM004, we used an *hcp hcp1* double mutant of FQ-A001 (Δ*hcp* Δ*hcp1*), which features deletion of the two *hcp* genes that encode identical Hcp subunits [9] and fails to form TssB sheaths (Fig. S2). We found that the abundance of erm^R^ ANM004 did not change when co-incubated with FQ-A001 but increased with the Δ*hcp* Δ*hcp1* strain (Fig. 3b), suggesting that T6SS2 activity in FQ-A001 has an inhibitory effect on ANM004 growth under these experimental conditions. Collectively, the findings suggest that both strains engage in interference competition against one another, with T6SS2 of ANM004 exhibiting a larger impact on the intercellular interactions between FQ-A001 and ANM004.

Previous work demonstrated that the quorum-sensing regulator LitR inhibits T6SS2 expression in FQ-A001 during interactions with ES114 [7](Fig. 4a). To determine whether LitR also modulates T6SS-mediated aggression of FQ-A001 towards ANM004, we performed co-incubation assays using SWTO medium, which enhances quorum-sensing traits in *V. fischeri* [16]. Relative to FQ-A001 cells grown to low cell density (LCD; OD_600_=0.20), cells grown to HCD (OD_600_=2.0) led to more ANM004 c.f.u. (Fig. 4b), indicating FQ-A001 aggression is lower at HCD. A Δ*litR* strain of FQ-A001 resulted in fewer ANM004 at later times, suggesting enhanced aggression towards ANM004 in the absence of LitR irrespective of cell density (Fig. 4b). Expression of *litR in trans* in the Δ*litR* mutant restored WT activity under either level of cell density (Fig. 4b), demonstrating genetic complementation. Together, these results indicate that LitR negatively regulates FQ-A001 aggression and that the quorum-sensing pathway influences competitive interactions between T6SS2^+^ strains.

**Fig. 4.**
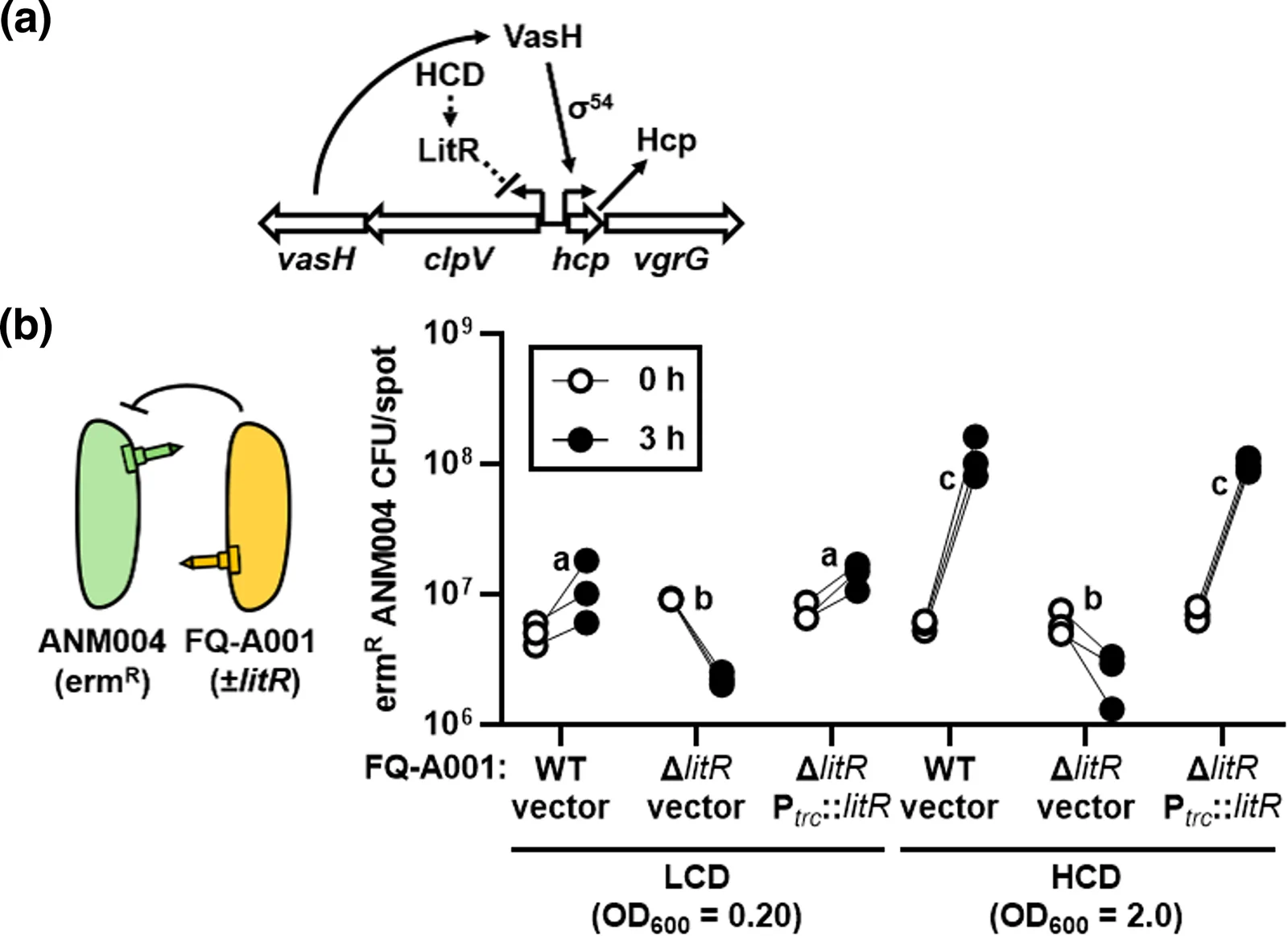
Quorum-sensing factors control FQ-A001 aggression towards ANM004. (**a)** Repression of VasH-dependent transcription of T6SS2 Hcp by LitR at HCD. (**b)** Abundance of TIM426 (erm^R^ ANM004) grown overnight in LBS co-incubated with FQ-A001 (WT) or KRG001 (Δ*litR*) harbouring pTM214 (vector) or pKRG023 (P*_trc_::litR*) grown in SWTO medium supplemented with 0.1 mM IPTG to LCD or HCD (with OD_600_=0.20 or 2.0, respectively) at indicated time points. *N*=4. Different letters indicate groups with statistically different log-transformed fold changes in abundance over time (*P*<0.05, adjusted for multiple comparisons) based on two-way ANOVA.

### Bioinformatic comparison of ANM004 and FQ-A001

Results from the co-incubation assays described above indicate that ANM004 and FQ-A001 exhibit T6SS2-dependent aggression towards each other. Comparison of their T6SS2 main and auxiliary gene clusters shows that gene synteny and sequence homology are generally conserved across loci (Fig. 5a and Table 3), with three notable exceptions. First, the genes encoding putative PAAR and AHH nuclease proteins in FQ-A001 are separate genes (*VFFQA001_15490* and *VFFQA001_15495*) but occur as a fused gene (*AC1TBE_15750*) in ANM004. Second, immediately downstream of *AC1TBE_15750* is an 18-gene locus that disrupts the synteny observed in FQ-A001 and encodes at least seven ORFs with no detectable homology to proteins encoded by FQ-A001. Finally, auxiliary gene clusters 1 and 3 in ANM004 contain multiple ORFs that appear as single genes in the corresponding clusters of FQ-A001.

**Fig. 5.**
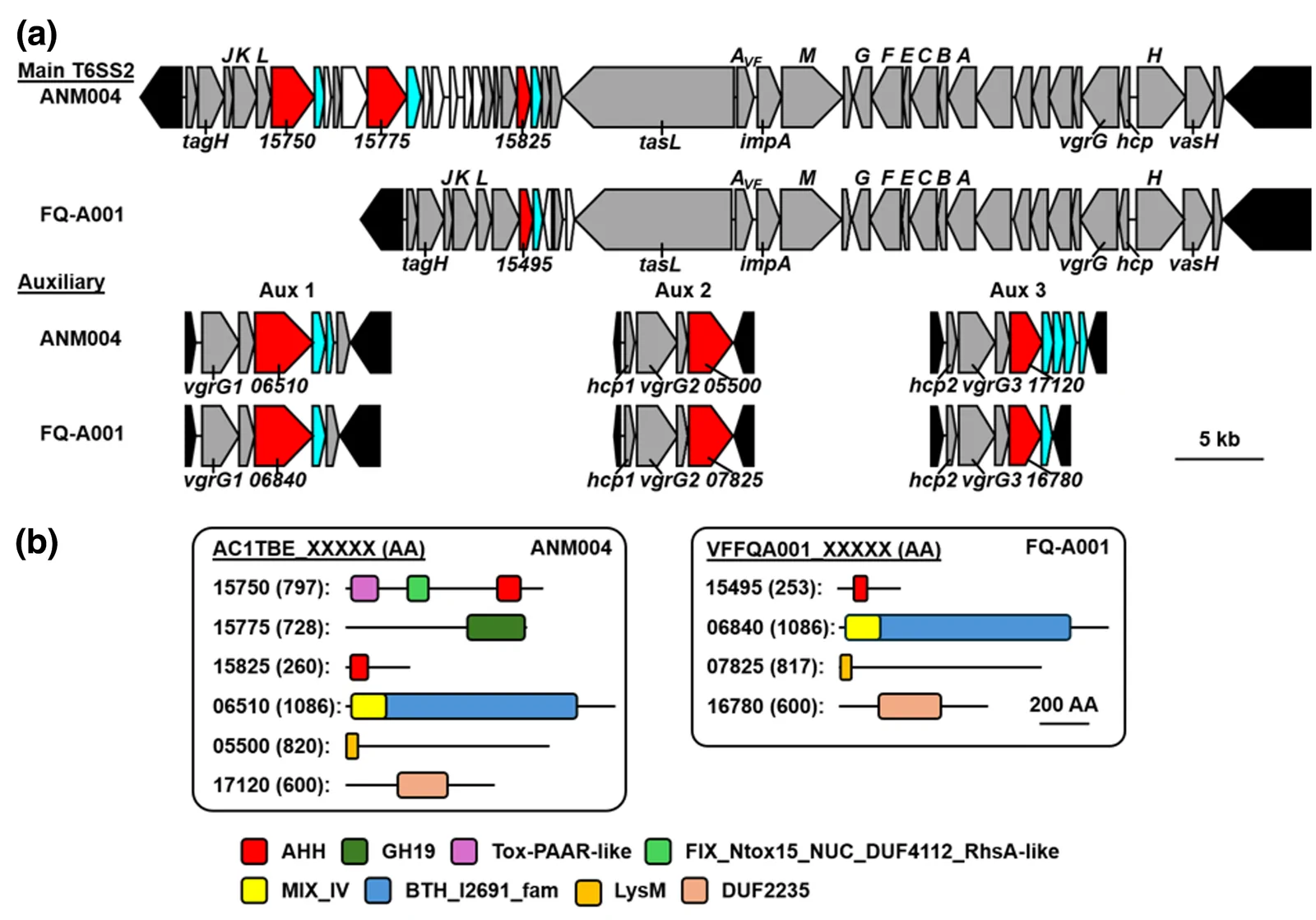
Putative effectors within T6SS gene clusters of ANM004 and FQ-A001. (**a)** Main T6SS2 (above) and auxiliary (below) gene clusters of ANM004 (top) and FQ-A001 (bottom). Putative effectors and immunity proteins are shown in red and cyan, respectively. Other genes that share homology between strains are shown in grey. White genes indicate genes with low identity or are unique to individual strains. Gene names shown as individual letters have prefix *tss*, and those with numbers have prefix AC1TBE_ (ANM004) or VFFQA001_RS (FQ-A001). (**b)** Domains of putative T6SS effectors in ANM004 (left) and FQ-A001 (right). Protein IDs are shown with size (in AA) shown in parentheses.

**Table 3.** ANM004 T6SS2 genes and homologs in FQ-A001

Locus tag	Accession	Common name	Short name	FQ-A001 Homologue (% identity)*
**Main T6SS2 gene cluster**				
*AC1TBE_15725*	None detected		Hypothetical protein	VFFQA001_RS15465 (100)
*AC1TBE_15730*	COG3456	*tagH*	COG3456	VFFQA001_RS15470 (98.4)
*AC1TBE_15735*	TIGR03352	*tssJ*	VI_chp_3	VFFQA001_RS15475 (100)
*AC1TBE_15740*	COG3522	*tssK*	COG3522	VFFQA001_RS15480 (99.8)
*AC1TBE_15745*	COG3455	*tssL*	DotU	VFFQA001_RS15485 (99.2)
*AC1TBE_15750*	pfam13665 cd20746 pfam14412		Tox-PAAR-like FIX_Ntox15_NUC_DUF4112_RhsA-like AHH	VFFQA001_RS15490 (54.6) VFFQA001_RS15495 (34.5)
*AC1TBE_15755*	None detected		Hypothetical protein	Unique to ANM004
*AC1TBE_15760*	None detected		Hypothetical protein	Unique to ANM004
*AC1TBE_15765*	cl01370	*tssL*	DotU superfamily	VFFQA001_RS15485 (97.7)
*AC1TBE_15770*	pfam13665		Tox-PAAR-like	VFFQA001_RS15490 (44.5)
*AC1TBE_15775*	COG3179		GH19	Unique to ANM004
*AC1TBE_15780*	None detected		Hypothetical protein	Unique to ANM004
*AC1TBE_15785*	None detected		Hypothetical protein	Unique to ANM004
*AC1TBE_15790*	None detected		Hypothetical protein	VFFQA001_RS15500 (23.1)
*AC1TBE_15795*	None detected		Hypothetical protein	VFFQA001_RS15510 (40.0)
*AC1TBE_15800*	None detected		Hypothetical protein	Unique to ANM004
*AC1TBE_15805*	None detected		Hypothetical protein	Unique to ANM004
*AC1TBE_15810*	None detected		Hypothetical protein	VFFQA001_RS15510 (90.8)
*AC1TBE_15815*	None detected		Hypothetical protein	VFFQA001_RS15485 (60.3)
*AC1TBE_15820*	cd20746		FIX_Ntox15_NUC_DUF4112_RhsA-like	VFFQA001_RS15490 (91.1)
*AC1TBE_15825*	pfam14412		AHH	VFFQA001_RS15495 (29.1)
*AC1TBE_15830*	None detected		Hypothetical protein	VFFQA001_RS15500 (27.0)
*AC1TBE_15835*	None detected		Hypothetical protein	VFFQA001_RS15510 (90.8)
*AC1TBE_15840*	None detected		Hypothetical protein	VFFQA001_RS15510 (98.6)
*AC1TBE_15845*	NF032891 cl41065	*tle1*	tail_200_repeat Big_12 superfamily	VFFQA001_RS15520 (88.6)
*AC1TBE_15850*	cl36042	*mepM*	PRK11649 superfamily	VFFQA001_RS15525 (100)
*AC1TBE_15855*	pfam06812 cl49294	*impA*	ImpA_N IglI_fam superfamily	VFFQA001_RS15530 (99.5)
*AC1TBE_15860*	cl34628	*tssM*	IcmF superfamily	VFFQA001_RS15535 (99.8)
*AC1TBE_15865*	COG1522		Lrp	VFFQA001_RS15540 (100)
*AC1TBE_15870*	TIGR03347	*tssG*	VI_chp_1	VFFQA001_RS15545 (100)
*AC1TBE_15875*	TIGR03359	*tssF*	VI_chp_6	VFFQA001_RS15550 (99.8)
*AC1TBE_15880*	TIGR03357	*tssE*	VI_zyme	VFFQA001_RS15555 (99.3)
*AC1TBE_15885*	TIGR03355	*tssC*	VI_chp_2	VFFQA001_RS15560 (100)
*AC1TBE_15890*	COG3516	*tssB*	TssB	VFFQA001_RS15565 (100)
*AC1TBE_15895*	pfam16989 pfam06812	*tssA*	T6SS_VasJ ImpA_N	VFFQA001_RS15570 (99.4)
*AC1TBE_15900*	cl21453		PKc_like superfamily	VFFQA001_RS15575 (99.4)
*AC1TBE_15905*	None detected		Hypothetical protein	VFFQA001_RS15580 (98.7)
*AC1TBE_15910*	COG5351		COG5351	VFFQA001_RS15585 (99.4)
*AC1TBE_15915*	None detected		Hypothetical protein	VFFQA001_RS15590 (98.2)
*AC1TBE_15920*	None detected		Hypothetical protein	VFFQA001_RS15595 (98.8)
*AC1TBE_15925*	TIGR03361 cl33691	*vgrG*	VI_Rhs_Vgr five superfamily	VFFQA001_RS15600 (100)
*AC1TBE_15930*	TIGR03344	*hcp*	VI_effect_Hcp1	VFFQA001_RS15605 (100)
*AC1TBE_15935*	TIGR03345	*tssH*	VI_ClpV1	VFFQA001_RS16800 (99.8)
*AC1TBE_15940*	COG3829	*vasH*	RocR	VFFQA001_RS15615 (100)
*AC1TBE_15945*	smart00065		GAF	VFFQA001_RS15620 (99.4)
**Auxiliary gene cluster 1**				
*AC1TBE_06520*	cl37255	*vgrG*	VI_Rhs_Vgr superfamily	VFFQA001_RS06830 (98.2)
*AC1TBE_06515*	pfam13503	*tap*	DUF4123	VFFQA001_RS06835 (95.8)
*AC1TBE_06510*	cd20708		MIX_IV	VFFQA001_RS06840 (94.6)
*AC1TBE_06505*	cl49522	*tke5*	BTH_I2691_fam superfamily	VFFQA001_RS06845 (74.1)
*AC1TBE_06500*	cl43280	*tled1*	Lnt superfamily	VFFQA001_RS06845 (81.7)
*AC1TBE_06495*	None detected		Hypothetical protein	VFFQA001_RS06850 (96.6)
**Auxiliary gene cluster 2**				
*AC1TBE_05485*	TIGR03344	*hcp*	VI_effect_Hcp1	VFFQA001_RS15605 (99.4)
*AC1TBE_05490*	TIGR03361 cl24267	*vgrG*	VI_Rhs_Vgr Cthe_2159 superfamily	VFFQA001_RS07835 (98.7)
*AC1TBE_05495*	cl16292	*tap*	DUF4123 superfamily	VFFQA001_RS07830 (93.4)
*AC1TBE_05500*	cl21525		LysM superfamily	VFFQA001_RS07825 (93.2)
**Auxiliary gene cluster 3**				
*AC1TBE_17135*	TIGR03344	*hcp*	VI_effect_Hcp1	VFFQA001_RS16800 (100)
*AC1TBE_17130*	TIGR03361	*vgrG*	VI_Rhs_Vgr	VFFQA001_RS16795 (98.5)
*AC1TBE_17125*	pfam13503	*tap*	DUF4123	VFFQA001_RS16790 (96.2)
*AC1TBE_17120*	cl01480	*tle1*	DUF2235 superfamily	VFFQA001_RS16780 (87.2)
*AC1TBE_17115*	cl12703	*tli1*	DUF2931 superfamily	VFFQA001_RS16775 (78.5)
*AC1TBE_17110*	cl12703	*tli1*	DUF2931 superfamily	VFFQA001_RS16775 (79.2)
*AC1TBE_17105*	cl12703	*tli1*	DUF2931 superfamily	VFFQA001_RS16775 (77.6)
*AC1TBE_17100*	cl12703	*tli1*	DUF2931 superfamily	VFFQA001_RS16775 (95.9)

* Homologue with largest % identity is listed.

T6SS-dependent aggression depends on the delivery of effectors into other cells where they exhibit toxicity [2, 17]. The main and auxiliary T6SS2 gene clusters of ANM004 are predicted to encode at least six effectors (Fig. 5b and Tables 3 and 4). The three effectors encoded by the auxiliary gene clusters (AC1TBE_06510, AC1TBE_05500 and AC1TBE_17120) exhibit high homology (>90% identity) to the corresponding factors in FQ-A001 (Table 3), suggesting a limited role in driving the aggression displayed between ANM004 and FQ-A001. In contrast, the effectors encoded by the main T6SS2 gene cluster in ANM004 (AC1TBE_15750, AC1TBE_15775 and AC1TBE_15825) show low homology to factors encoded by FQ-A001 (Table 3), making them potential effectors underlying the T6SS2-dependent aggression ANM004 displays towards FQ-A001.

**Table 4.** Putative T6SS effectors encoded by ANM004

Locus tag	Effector domain	Proposed function
AC1TBE_15750	AHH	Predicted nuclease of the HNH/ENDO VII superfamily
AC1TBE_15775	GH19	Putative chitinase, peptidoglycan ld-endopeptidase
AC1TBE_15825	AHH	Predicted nuclease of the HNH/ENDO VII superfamily
AC1TBE_06510	MIX_IV	VasX toxin (VasX) family
AC1TBE_05500	LysM superfamily	Enzyme involved in bacterial cell wall degradation
AC1TBE_17120	DUF2235 superfamily	Uncharacterized α/β hydrolase

### AC1TBE_15825 is a T6SS2 effector that inhibits FQ-A001 growth

To begin testing whether AHH nucleases contribute to the aggression between *V. fischeri* strains, we selected the putative AHH nuclease encoded by *AC1TBE_15825* as a starting point. Cells typically avoid self-toxicity from an effector by co-expressing a cognate immunity factor encoded immediately downstream of the effector gene. Therefore, to determine whether FQ-A001 is sensitive to AC1TBE_15825, we constructed a plasmid featuring *AC1TBE_15825* and downstream gene *AC1TBE_15830* under IPTG-inducible control (P*_trc_*::AHH-IF) and introduced this plasmid into FQ-A001 (Fig. 6a). IPTG induction in FQ-A001 did not affect growth (Fig. S3). However, when FQ-A001 harbouring P*_trc_*::AHH-IF was co-incubated with erm^R^ FQ-A001 in the presence of IPTG, the growth of erm^R^ FQ-A001 stalled after 2 h (Fig. 6b), suggesting that induction of *AC1TBE_15825-30* expression in FQ-A001 can inhibit the growth of kin. However, the erm^R^ FQ-A001 grew normally when co-incubated with the Δ*hcp* Δ*hcp1* strain harbouring P*_trc_*::AHH-IF (Fig. 6c), suggesting that AC1TBE_15825-30-mediated inhibition of kin growth depends on T6SS2.

**Fig. 6.**
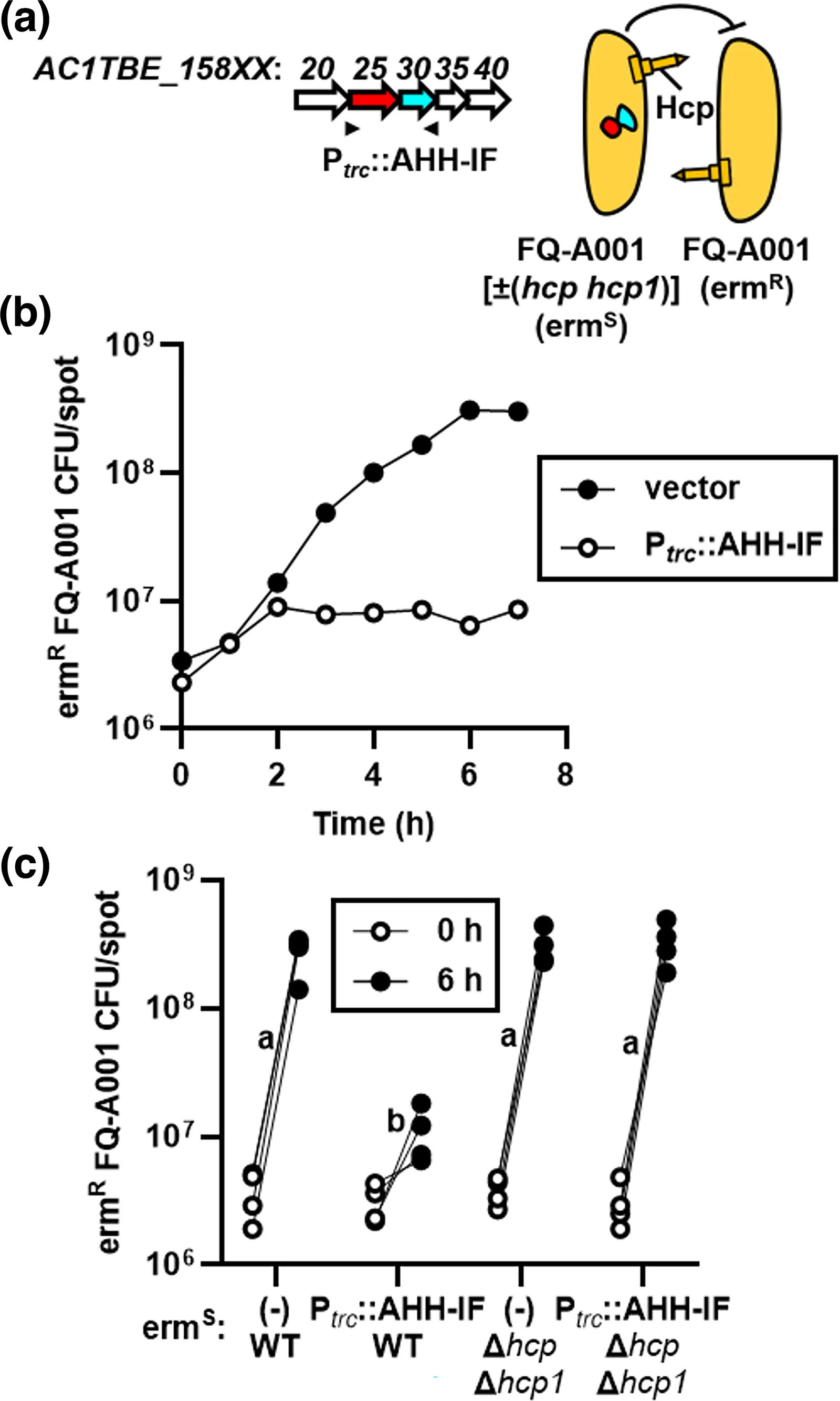
Inhibition of kin growth by ectopic expression of T6SS-dependent effector-immunity pair. (**a)***Left*, predicted operon encoding predicted AHH nuclease AC1TBE_15825 (AHH) and immunity factor AC1TBE_15825 (IF). Arrows indicate region amplified for IPTG-inducible construct (P*_trc_*::AHH-IF). *Right*, cartoon of model of T6SS-dependent interactions between erm^S^ FQ-A001 harbouring P*_trc_*::AHH-IF and erm^R^ FQ-A001. (**b)** Abundance of AGC020 (erm^R^ FQ-A001) incubated with FQ-A001 harbouring pAEM002 (P*_trc_*::AHH-IF) or pTM214 (vector). (**c)** Abundance of AGC020 (erm^R^ FQ-A001) incubated with FQ-A001 (WT) or NPW58 (Δ*hcp* Δ*hcp1*) harbouring pAEM002 (P*_trc_*::AHH-IF) or pTM214 (-). *N*=4. Different letters indicate groups with statistically different log-transformed fold changes in abundance over time (*P*<0.05, adjusted for multiple comparisons), from one-way ANOVA (F_3,12_=34.34, *P*<0.0001).

AHH nucleases belong to the HNH/ENDOVII superfamily of Zn^2+^-binding proteins characterized by treble-clef folds [18]. The AHH name reflects a conserved motif (SHH in AC1TBE_15825), with the two histidine residues predicted to interact with a Zn^2+^ ion and facilitate ATP hydrolysis, respectively. Structural modelling with AlphaFold 3.0 identified three histidine residues (H37, H87 and H91) that coordinate a Zn^2+^ ion, along with another histidine residue (H38) positioned to play a catalytic role (Fig. S4). To determine whether this residue is required for AC1TBE_15825-30-mediated inhibition of kin growth, we conducted co-incubation assays using FQ-A001 expressing an H38A variant of AC1TBE_15825. In contrast to the WT control, the H38A variant permitted growth of erm^R^ FQ-A001 (Fig. 7a), indicating that H38 is necessary for inhibition of kin growth.

**Fig. 7.**
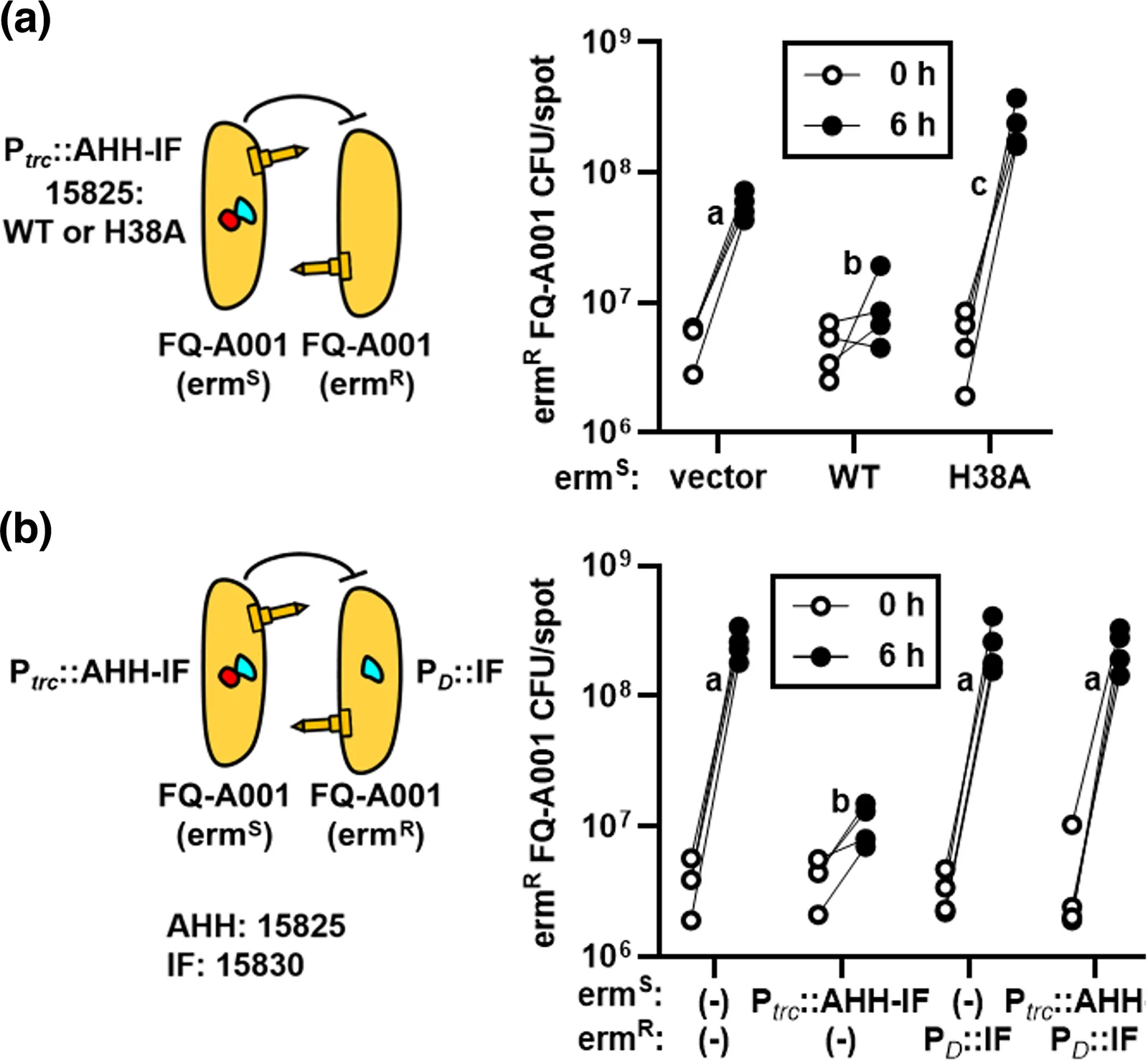
Genetic analysis of AC1TBE_15825-30-mediated kin growth inhibition. (**a)***Left*, model for assessing contribution of H38 to AC1TBE_15825 activity. *Right*, abundance of AGC020 (erm^R^ FQ-A001) incubated with FQ-A001 (erm^S^) harbouring pAEM002 (WT), pKRG055 (H38A) or pTM214 (vector). *N*=4. One-way ANOVA (F_2,9_=20.12, *P*=0.0005) (**b)** Abundance of AGC020 (erm^R^ FQ-A001) with either pTM447 (P*_D_*::IF) or pSCV51 (-) incubated with FQ-A001 (WT) harbouring pAEM002 (P*_trc_*::AHH-IF) or pTM214 (-). AHH=AC1TBE_15825; IF=AC1TBE_15830. n=4. One-way ANOVA (F_3,12_ = 54.00, *P*<0.0001). Different letters indicate groups with statistically different log-transformed fold changes in abundance over time (*P<*0.05), with *P*-values adjusted for multiple comparisons.

To test whether AC1TBE_15830 confers immunity to *AC1TBE_15825-30*-dependent growth inhibition, we modified the experiment so that erm^R^ FQ-A001 carried a plasmid that constitutively expresses *AC1TBE_15830* (P*_D_::AC1TBE_15830*). In contrast to the vector control, erm^R^ FQ-A001 P*_D_::AC1TBE_15830* grew during co-incubation with FQ-A001 P*_trc_::AC1TBE_15825-30* (Fig. 7b), indicating that expression of *AC1TBE_15830* protects against AC1TBE_15825-30-mediated growth inhibition of kin. Structural modelling by AlphaFold 3.0 further supports this interpretation, predicting with very high confidence (pLDDT >90) that AC1TBE_15825 and AC1TBE_15830 form a protein–protein interface (Fig. S5), consistent with a mechanism in which AC1TBE_15830 directly inhibits AC1TBE_15825 activity. Together, these results indicate that FQ-A001 is susceptible to intoxication by T6SS-delivered AC1TBE_15825, whereas AC1TBE_15830 functions as the cognate immunity protein that protects against this activity.

## Discussion

The ability of bacteria to influence the fitness of neighbouring cells within a shared habitat plays a critical role in shaping the structure and function of microbial ecosystems. The T6SS is a prominent example of a contact-dependent mechanism widely distributed across diverse environments, including animal tissues, where microbial communities contribute to host fitness and behaviour. Elucidating how T6SS activity impacts host-associated microbes has the potential to reveal new ways to manipulate community composition within tissues, thereby offering novel approaches to modulate host physiology.

In this study, we report ANM004 as a novel symbiotic, T6SS2^+^ strain of *V. fischeri* that serves as a source of effectors that inhibit the growth of other strains, such as FQ-A001. ANM004 encodes putative T6SS2 effectors not found in FQ-A001, and they are predicted to target either nucleic acids or peptidoglycan. While the suite of effectors that promote ANM004 aggression toward FQ-A001 remain unclear, one potential candidate is the AHH nuclease encoded by *AC1TBE_15825*, which inhibits FQ-A001 growth. T6SS2-mediated kin growth inhibition by AC1TBE_15825 is prevented by its cognate immunity factor AC1TBE_15830.

Taken together, these results suggest that the outcome of interactions between two T6SS2^+^ *V. fischeri* strains largely depends on the effector payload and susceptibility profile of each strain. While only one toxin was experimentally examined in this study, bioinformatics analysis suggests both FQ-A001 and ANM004 encode multiple unique effectors. A recent study of T6SS in *Acinetobacter baylyi* coupling modelling and experimental evolution revealed that deploying multiple distinct effectors is a more effective strategy to limit resistance evolution in competitors than a single toxin, which can permit the evolution of cross-resistance to other types of toxins [19]. Therefore, the presence of multiple unique toxins in FQ-A001 and ANM004 suggests that T6SS2-mediated antagonism may be an important driver of *V. fischeri* strain divergence. Furthermore, the greater number of putative effectors encoded by ANM004 may contribute to its competitive dominance over FQ-A001 during co-incubation (Fig. 3). However, the specificity of individual effectors, such as AC1TBE_15825, as well as strain-specific transcriptional regulation of the T6SS2 by factors such as LitR, are also likely to shape interactions between strains. Consequently, determining strain susceptibility or resistance to additional effectors, such as the GH19-domain containing factor AC1TBE_15775, will help elucidate how T6SS-mediated interactions influence the evolutionary trajectories of T6SS2^+^ *V. fischeri* strains.

The immunity factors for T6SS effectors are frequently encoded directly downstream of effector genes, and co-expression prevents self-intoxication. A recent report demonstrated that the immunity factors encoded by *Myxococcus xanthus* can also interact to different extents with noncognate toxins, resulting in strains exhibiting varying degrees of antagonism and, in some cases, even cooperation [20]. Despite FQ-A001 likely encoding a putative immunity factor (15500) against an AHH-domain containing protein (15495), it does not appear to confer immunity against AC1TBE_15825. However, the observation of several genes encoding homologous hypothetical proteins within the T6SS2 loci of ANM004, e.g. *AC1TBE_17100-115* in Auxiliary Cluster 3, points to gene duplication and diversification events that may contribute to complex compatibility rules among different strains.

The ability of FQ-A001 to inhibit the growth of an otherwise isogenic strain using an effector derived from ANM004 (Fig. 6) suggests a high degree of functional compatibility between the T6SSs of these strains. Similar compatibility has been observed in other systems, *e.g.*, expression of the lipase effector TleV1 from one *Vibrio cholerae* strain in another was sufficient to promote kin killing [21]. However, heterologous effectors do not always function in recipient strains, as their secretion can depend on specific adaptors or chaperones that facilitate effector loading onto the T6SS [22–24]. The structural model of AC1TBE_15825 suggests that most of the polypeptide adopts a nuclease fold (Fig. S4), providing an opportunity for future studies to identify the surface features that mediate its recognition and loading by the T6SS. Such studies could help define the molecular determinants governing effector compatibility and the exchange of T6SS effectors between *V. fischeri* strains.

As illustrated by the results shown in Fig. 4, the extent to which *V. fischeri* exhibit aggression towards other cells is also influenced by quorum sensing, indicating that environmental factors are critical for understanding whether ecological strategies based on T6SS2, like interference competition, are executed within a specific ecosystem. For instance, during initial colonization of the squid light organ, only a few cells will enter each crypt space. Consequently, each population will initially be at LCD, which is an environmental factor known to inhibit LitR expression, thereby permitting T6SS2 expression and interference competition [7]. As the population grows, the cells engage in quorum sensing, leading to LitR repressing T6SS2 expression and promoting bioluminescence production. How repression of T6SS2 gene expression by LitR inhibits T6SS2 activity is unclear, with T6SS2 assembly, firing, and disassembly representing specific points within the cycle that could be affected by regulation. Because the T6SS2 gene clusters of FQ-A001 and ANM004 lack homologs of *pppA* ([3] and Table 3, respectively), it is unlikely that these strains use the threonine phosphorylation pathway that underlies the ‘tit-for-tat’ defensive strategy observed in *Pseudomonas aeruginosa* [25]. Regardless, our results with the two-strain model described here suggest that the interactions that take place between different T6SS2^+^ strains are highly dynamic. Furthermore, such competition among different strains may stabilize population structures in a way that maintains strain diversity [26]. Future work will examine these interactions *in vivo* as the populations switch from competitive strategies to collaborative ones.

In summary, this study demonstrates reciprocal aggression between distinct T6SS2^+^ *V. fischeri* strains, thereby establishing a tractable experimental framework for investigating toxic effector-immunity pairs in this bacterial symbiont. Although our analysis is limited to two strains, the findings indicate that T6SS2 activity by each strain drives highly dynamic, bidirectional interactions. Future studies should expand this framework to more complex strain ensembles and *in vivo* contexts to determine how such interactions shape the structure and function of symbiotic populations within the host, ultimately clarifying the role of bacterial symbionts in modulating host physiology.

## Supplementary material

10.1099/mic.0.001781 Fig. S1.

## Abbreviations

FSSW: filter-sterilized seawater
HCD: high cell density
LBS: Luria-Bertani salt
LCD: low cell density
SWTO: seawater-tryptone with osmolarity
T6SS: type VI secretion system
