# Supplementary material for: Type VI secretion drives reciprocal growth inhibition among symbiotic Vibrio fischeri strains
Source: Microbiology (Reading). 2026 Oct 23;172(10):001781. doi: 10.1099/mic.0.001781 (PMC13694042; doi:10.1099/mic.0.001781)
Supplement: Supplementary file 1 [file mic-172-01781-s001.pdf]

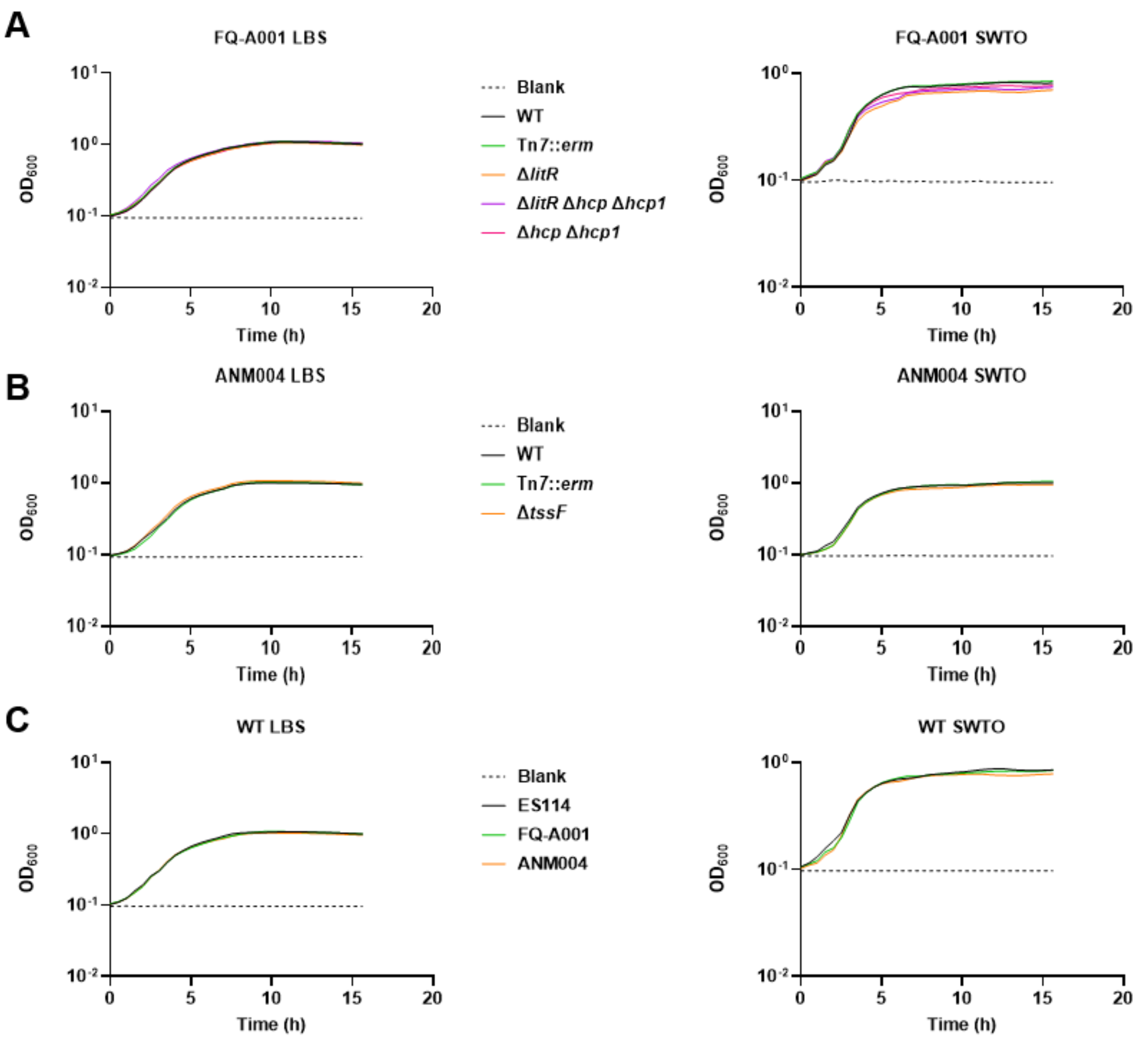

Fig. S1: Growth curves of *V. fischeri* strains used in this study.
Turbidity of indicated strains grown in LBS (*left*) or SWTO (*right*) over time.
A. FQ-A001 (WT), AGC020 (Tn*7*::*erm*), KRG001 (Δ*litR*), KRG014 (Δ*litR* Δ*hcp* Δ*hcp1*), and NPW58 (Δ*hcp* Δ*hcp1*).
B. ANM004 (WT), TIM426 (Tn*7*::*erm*), and KRG017 (Δ*tssF*).
C. ES114, FQ-A001, and ANM004.
Dotted lines indicate a blank media control.

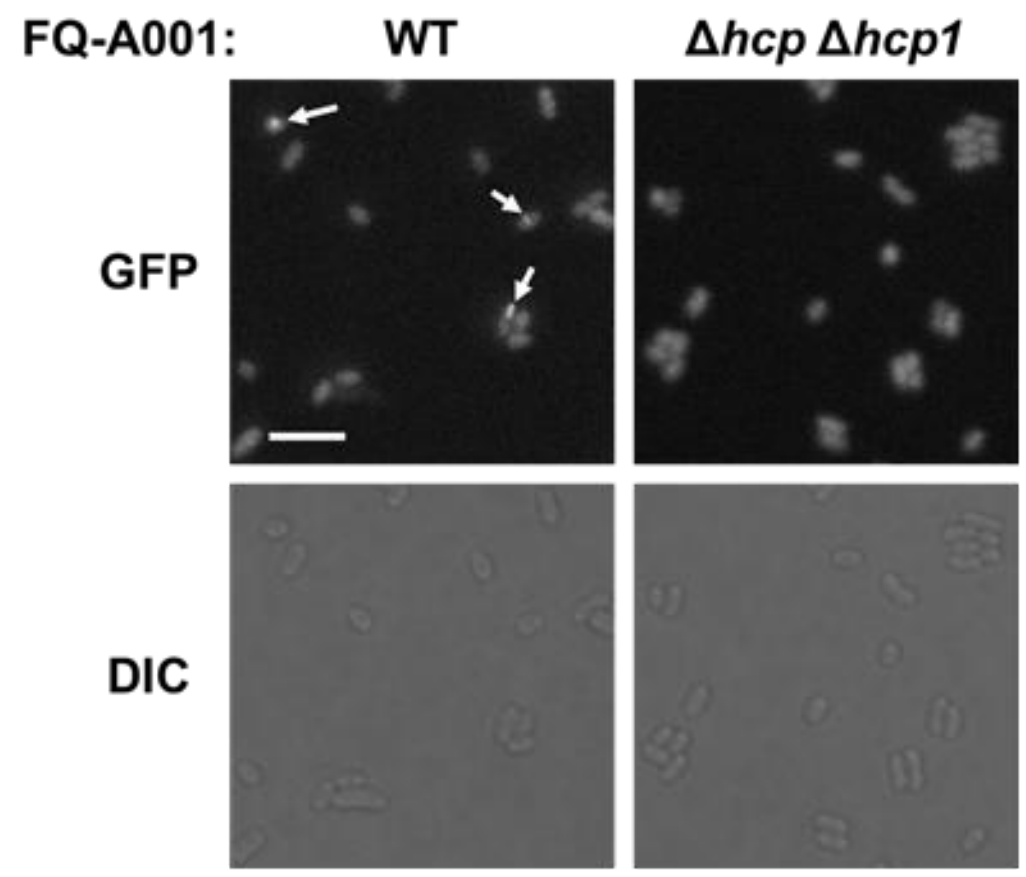

Fig. S2: T6SS2 assembly in FQ-A001
TssB::GFP localization pattern in FQ-A001 (WT) and NPW58 (Δ*hcp* Δ*hcp1*) harboring pSNS119 plasmid. Arrows point to examples of subcellular GFP puncta. Scale bar = 5 µm.

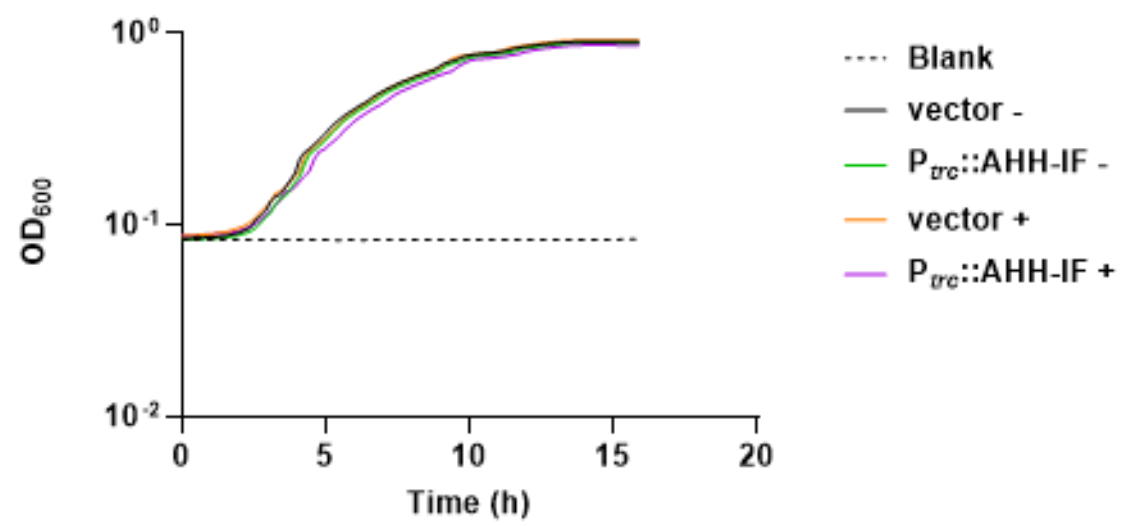

Fig. S3: Growth curves of *V. fischeri* strain FQ-A001 harboring pTM214 (vector) or pAEM002 (P_{trc}::AHH-IF) in LBS supplemented with (+) or without (-) IPTG. Dotted line indicates a blank media control.

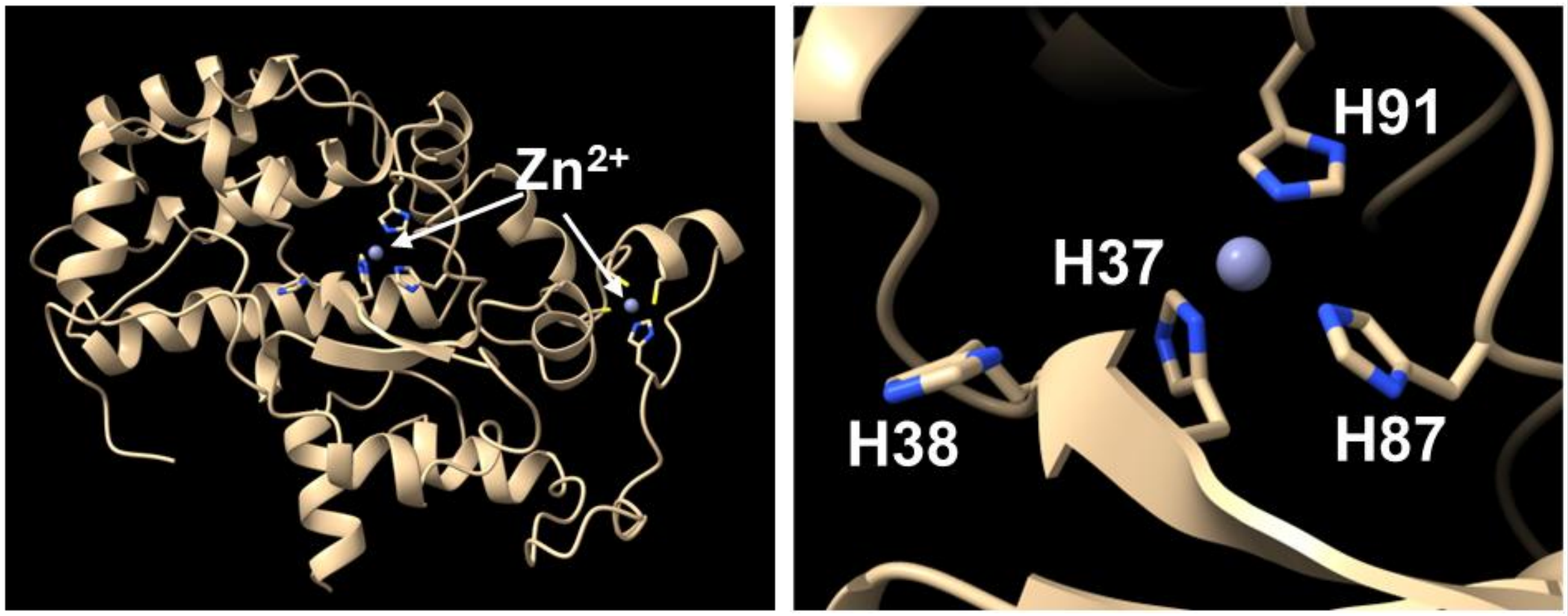

Fig. S4: Model of AC1TBE_15825 structure

Left, Structural model of AC1TBE_15825 predicted by AlphaFold 3.0. Arrows point to the two Zn^{2+} ions that are coordinated by the residues that are shown. *Right*, view of histidine residues (H37, H91, & H87) that coordinate a Zn^{2+} ion and the predicted catalytic histidine residue H38.

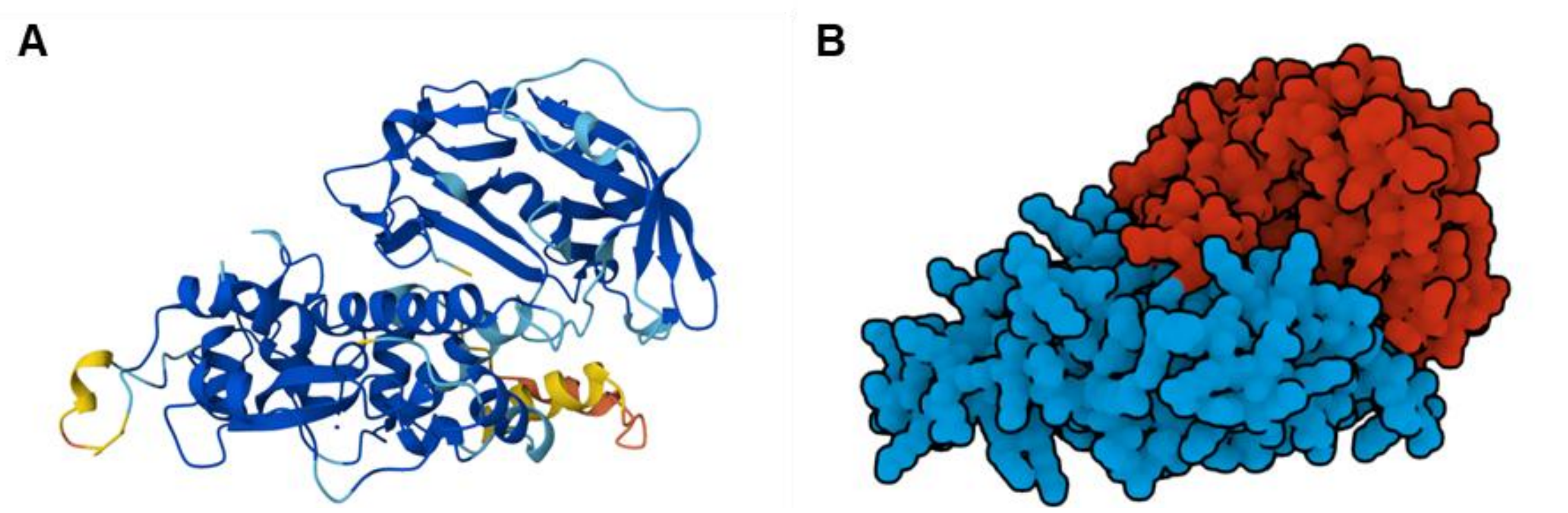

Fig. S5: Interaction model of AC1TBE_15825-30

A. Structural model of AC1TBE_15825 (with two Zn^{2+} ions) and AC1TBE_15830 predicted by AlphaFold 3.0. Colors indicate pIDDT levels, with blue (pIDDT > 90), cyan (90 > pIDDT > 70), yellow (70 > pIDDT > 50), and orange (pIDDT < 50).

B. Space-filling model of predicted structure shown in A, with AC1TBE_15825 and AC1TBE_15830 shaded in blue and red, respectively.
